# General Anesthetic Agents and Renal Function after Nephrectomy

**DOI:** 10.3390/jcm8101530

**Published:** 2019-09-24

**Authors:** Ho-Jin Lee, Jinyoung Bae, Yongsuk Kwon, Hwan Suk Jang, Seokha Yoo, Chang Wook Jeong, Jin-Tae Kim, Won Ho Kim

**Affiliations:** 1 Department of Anesthesiology and Pain Medicine, Seoul National University Hospital, Seoul National University College of Medicine, Seoul 03080, Koreabaejy88@gmail.com (J.B.); ulsan-no8@hanmail.net (Y.K.); luminsishs@snu.ac.kr (H.S.J.); muroki22@gmail.com (S.Y.);; 2 Department of Urology, Seoul National University Hospital, Seoul National University College of Medicine, Seoul 03080, Korea; drboss@korea.com

**Keywords:** nephrectomy, acute kidney injury, chronic kidney disease, sevoflurane, desflurane, propofol

## Abstract

The association between the choice of general anesthetic agents and the risk of acute kidney injury (AKI) and long-term renal dysfunction after nephrectomy has not yet been evaluated. We reviewed 1087 cases of partial or radical nephrectomy. The incidence of postoperative AKI, new-onset chronic kidney disease (CKD) and CKD upstaging were compared between general anesthetic agent groups (propofol, sevoflurane, and desflurane). Four different propensity score analyses were performed to minimize confounding for each pair of comparison (propofol vs. sevoflurane; propofol vs. desflurane; sevoflurane vs. desflurane; propofol vs. volatile agents). Study outcomes were compared before and after matching. Kaplan-Meier survival curve analysis was performed to compare renal survival determined by the development of new-onset CKD between groups up to 36 months after nephrectomy. Propofol was associated with a lower incidence of AKI (propofol 23.2% vs. sevoflurane 39.5%, *p* = 0.004; vs. propofol 21.0% vs. desflurane 34.3%, *p* = 0.031), a lower incidence of CKD upstaging (propofol 27.2% vs. sevoflurane 58.4%, *p* < 0.001; propofol 32.4% vs. desflurane 48.6%, *p* = 0.017) and better three-year renal survival after nephrectomy compared to sevoflurane or desflurane group (Log-rank test propofol vs. sevoflurane *p* < 0.001; vs. desflurane *p* = 0.015) after matching. Propofol was also associated with a lower incidence of new-onset CKD after nephrectomy compared to sevoflurane after matching (*p* < 0.001). There were no significant differences between sevoflurane and desflurane. However, subgroup analysis of partial nephrectomy showed a significant difference only in CKD upstaging. In conclusion, propofol, compared to volatile agents, could be a better general anesthetic agent for nephrectomy to attenuate postoperative renal dysfunction. However, limitations of the retrospective study design and inconsistent results of the subgroup analysis preclude firm conclusions.

## 1. Introduction 

Kidney cancer, more than 90% of which is renal cell carcinoma (RCC), is common in both men and women [1]. Although partial or radical nephrectomy is the standard treatment for localized RCC [2], postoperative acute kidney injury (AKI) remains a common complication with a risk of evolving chronic kidney disease (CKD) [3,4] and the distant organ dysfunction [5]. Postoperative AKI and CKD after nephrectomy result in the prolonged length of hospital stay, increased medical cost and mortality [4,6,7]. Since acute postoperative renal dysfunction is associated with other delayed morbidities, it would be important to identify and correct potentially reversible risk factors of AKI [8]. 

Previous studies investigated perioperative predictors for AKI and CKD after nephrectomy [9,10,11,12]. However, to our knowledge, previously reported risk factors were generally not modifiable except ischemic time during renal arterial clamping, cold ischemia during partial nephrectomy, and intraoperative hypotension [9,13,14,15]. Effective interventions to decrease the risk of renal functional decline after nephrectomy are still required [16]. As another modifiable risk factor, the choice of general anesthetic agents would be important. General anesthetic agents may affect the postoperative renal function by the following mechanisms. Propofol, a widely used intravenous anesthetic agent, could prevent renal ischemia/reperfusion injury by anti-oxidative effect and progression of renal fibrosis by downregulating inducible nitric oxide synthase expression [17,18]. Although there were concerns regarding compound A-associated nephrotoxicity, many previous studies demonstrated the safety of sevoflurane. [19]. Conversely, sevoflurane had a protective effect on acute renal injury due to its anti-inflammatory effect in a previous animal study [20]. Therefore, propofol or sevoflurane may be associated with better postoperative renal function compared to other general anesthetics after nephrectomy. However, there have been no previous reports regarding the effect of general anesthetic agents on the postoperative renal function and it is unknown whether the choice of general anesthetic agents influences the risk of AKI or long-term renal function after partial or radical nephrectomy. 

Therefore, we attempted to investigate the association between the choice of general anesthetic agents and the risk of AKI and long-term renal function after nephrectomy [21]. We hypothesized that the incidences of AKI and new-onset CKD after general anesthesia with propofol may be lower than the incidences with sevoflurane or desflurane. To this aim, we conducted a retrospective cohort study to investigate the potential association between different anesthetic agents and the incidences of AKI and new-onset CKD after partial or radical nephrectomy. 

## 2. Materials and Methods

### 2.1. Study Design

This retrospective observational study was approved by the institutional review board (IRB) of Seoul National University Hospital (1905-089-1034). The requirement for written informed consent was waived by the IRB due to the retrospective design of this study. Studies were conducted in accordance with the approved guidelines and regulations.

### 2.2. Data Collection

After approval from the IRB, we reviewed the electronic medical records of 1087 adult patients underwent radical or partial nephrectomy due to a renal mass at our hospital between 2010 and 2014. Demographic or perioperative variables known to be associated with AKI or CKD after nephrectomy were collected (Table 1) [9,10,12]. The cohort was divided into three groups according to the anesthetic agents commonly used for maintenance of general anesthesia; propofol, sevoflurane, and desflurane. The patients who received agents other than these (*n* = 0) or whose main agent was changed during surgery (*n* = 0) or whose renal function after surgery was not followed up at least two times after surgery three months apart were excluded from our analysis (*n* = 0). 

### 2.3. Anesthesia and Surgical Techniques

The anesthetic protocols of our hospital during the study period were as follows. In the propofol group, general anesthesia was induced and maintained with a target-controlled infusion of propofol using infusion pump (Orchestra^®^; Fresenius Vial, Brezins, France). In the volatile agent groups, anesthesia was induced with propofol 1–2 mg/kg and maintained with either sevoflurane (2–4 vol %) or desflurane (5–7 vol %). In all groups, remifentanil was continuously infused throughout the surgery for balanced anesthesia, adjusted to maintain arterial pressure within 20% of baseline ward pressure. If arterial pressure was less than 20% of baseline despite adequate fluid administration and urine output, vasopressor including phenylephrine or norepinephrine was infused. The choice of anesthetic agents for anesthetic maintenance was made according to the anesthesiologists’ discretion. The decision was made according to the attending anesthesiologist’s preference regardless of patients’ comorbidity or baseline medical status. Patients were mechanically ventilated with a volume-controlled mode with a tidal volume of 6–8 mL/kg and a FiO_2_ of 0.4 to 0.5. Nephrectomies were conducted by open, laparoscopic, or robot-assisted techniques. Decisions regarding the type of surgical approach were made based on tumor characteristics. For partial nephrectomy, surgical resection was performed after clamping the main renal artery or arteries. The renal vein was clamped selectively. Saline ice slush was used for cold ischemia. Mannitol was administered intraoperatively within 30 min prior to renal vascular clamping.

### 2.4. Outcome Variables

The primary outcome of our study was the incidence of AKI after nephrectomy. Postoperative AKI was diagnosed by the Kidney Disease: Improving Global Outcomes (KDIGO) criteria, which was determined by the maximal change of the serum creatinine level during the first seven postoperative days (Stage 1: 1.5–1.9; stage 2: 2–2.9; stage 3: More than 3-fold increase from baseline) [22,23]. The most recent preoperative serum creatinine level was defined as the baseline value. 

The secondary outcomes included the incidence of new-onset CKD stage 3a or high (eGFR < 60 mL/min/1.73 m^2^), CKD upstaging after nephrectomy, the incidence of postoperative complications, and length of hospital stay. Postoperative new-onset CKD was diagnosed by the creatinine criteria of KDIGO criteria, which was determined when the estimated glomerular filtration rate (eGFR) decreased below 60 mL/min/1.73 m^2^ for three months or more [24]. We calculated eGFR from serum creatinine level using the Modification of Diet in Renal Disease (MDRD) study equation [25]. The most recent preoperative eGFR was defined as the baseline value. CKD upstaging was determined when the CKD stage follow-up was higher than the baseline until 3 years after nephrectomy.

### 2.5. Statistical Analysis

Statistical analyses were performed using SPSS software version 25.0 (IBM Corp., Armonk, NY, USA) and MedCalc Statistical Software version 18.6 (MedCalc Software bvba, Ostend, Belgium). A *p*-value of less than 0.05 was considered statistically significant. The Kolmogorov-Smirnov test was performed to determine the normality of the continuous variables. Continuous data are described as the mean (SD) or median (25 and 75 percentiles) and were compared by the independent *t*-test or the Mann-Whitney *U* test or one-way analysis of variance (ANOVA). In the pairwise comparisons between two anesthetic groups, Bonferroni correction was used by dividing the critical *p*-value by the number of comparisons to minimize the chance of a type 1 error. *p*-value < 0.017 was considered statistically significant. Categorical data are described as number (%) and were compared by the chi-square test or Fisher’s exact test. Missing data were less than 5% of the total records. We used simple imputation with median and mode. Missing values of continuous variables were replaced by the age-and sex-specific median values, and incidence data were assigned the most frequent age and sex-specific modes. The followings are main analyses of our study to evaluate the association between the general anesthetic agents and clinical outcomes. 

Firstly, to reduce the influence of confounding variables, four different propensity score matching analyses were performed to adjust for intergroup differences; Propofol vs. Sevoflurane, Propofol vs. Desflurane, Sevoflurane vs. Desflurane, and Propofol vs. volatile agents. The following variables were used as contributors to the propensity score: Sex, age, body-mass index, current smoking, history of hypertension, diabetes mellitus, cerebrovascular disease, chronic hepatitis or cirrhosis, ischemic heart disease, dyslipidemia, preoperative hemoglobin, serum albumin, eGFR, TNM stage of renal cell carcinoma, year of surgery, open surgery (vs. laparoscopic surgery), radical nephrectomy (vs. partial nephrectomy), operation time, unit number of packed red cell transfusion, crystalloid and colloid administration and need for vasopressor infusion. All patients were matched at a 1:1 ratio using the nearest neighbor method with a caliper width of 0.2 of the pooled standard deviation of the logit of the propensity score. To evaluate the balance of the matched patients, the standardized mean difference for each contributor was compared before and after matching. In each propensity-matched cohort, we directly compared the incidences of postoperative AKI and other secondary outcomes. 

Secondly, to evaluate the effect of general anesthetic agents on long-term renal function, Kaplan-Meier survival curve analyses were performed. Renal survival was determined by the development of new-onset CKD stage 3a or higher and the survival was compared between different anesthetic agent groups before and after matching. Patients were followed for up to 36 months and the log-rank test was used for inter-group comparison. 

Thirdly, we performed a subgroup analysis for the patients who underwent partial nephrectomy. We compared our primary and secondary outcomes between the propofol and volatile agent groups.

Although power calculation was not conducted prior to analysis, available power was calculated with the number of patients used in our analysis. With 130 and 644 patients used to compare the incidence of AKI between propofol and sevoflurane group and incidences of AKI of the two groups observed in our study, there was about 84.7% power to detect the observed difference in AKI. However, power decreased to 76.0% in the matched cohort between propofol and sevoflurane. 

## 3. Results 

**Figure 1:**
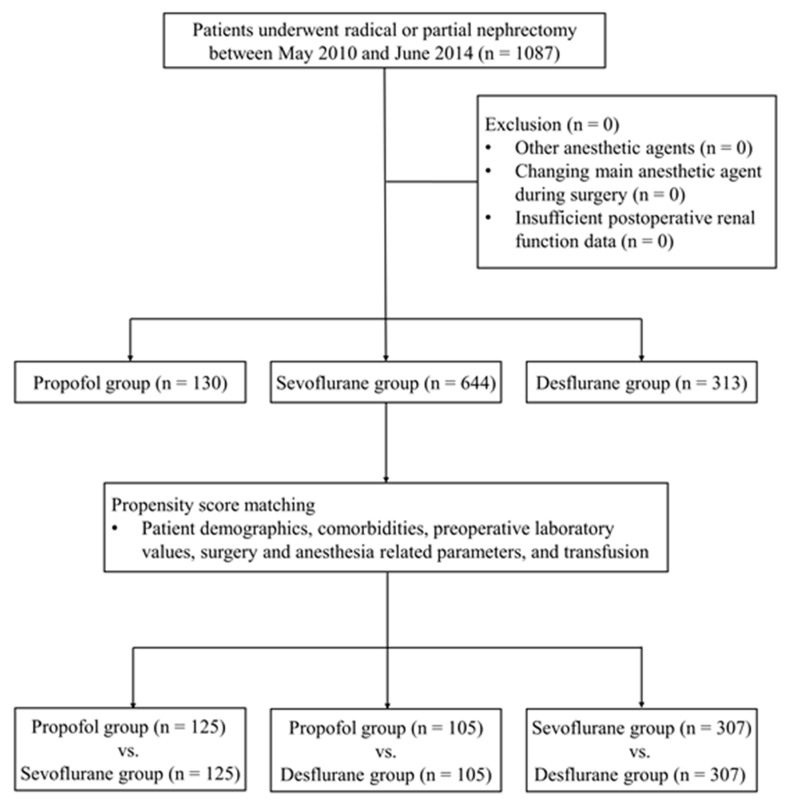
Flow diagram of the present study.

Among 1087 patients included in our analysis, 130 patients (12.0%) received propofol and 957 patients (88.0%: Sevoflurane 59.2%, Desflurane 28.8%) received volatile agent to maintain general anesthesia. After propensity score matching, 125 pairs of patients were matched between the propofol and sevoflurane group, 105 pairs between the propofol and desflurane group, and 307 pairs between the sevoflurane and desflurane group (Figure 1). Patient characteristics and perioperative parameters are summarized in Table 1. Histograms and covariate balance plots of the distribution of standardized differences of covariates between groups before and after matching are shown in Supplemental Figures S1–S4 according to the different pairs of matching.

There were significant differences in the incidences of postoperative AKI, new-onset CKD stage 3a or high and CKD upstaging between the propofol and volatile groups. (Table 2 and Table 3) However, there was no significant difference between the sevoflurane and desflurane groups (Table 4). After propensity score matching, the propofol group still showed significantly less frequent postoperative AKI, new-onset CKD stage 3a or high, and CKD upstaging than the sevoflurane group (Table 2). The propofol group also showed significantly less frequent postoperative AKI and CKD upstaging than the desflurane group (Table 3). Between sevoflurane and desflurane groups, there was no significant difference (Table 4). When the sevoflurane and desflurane groups were combined into the volatile group, the propofol group showed significantly less frequent postoperative AKI and CKD upstaging than the volatile group before and after matching (Supplemental Table S1).

**Figure 2:**
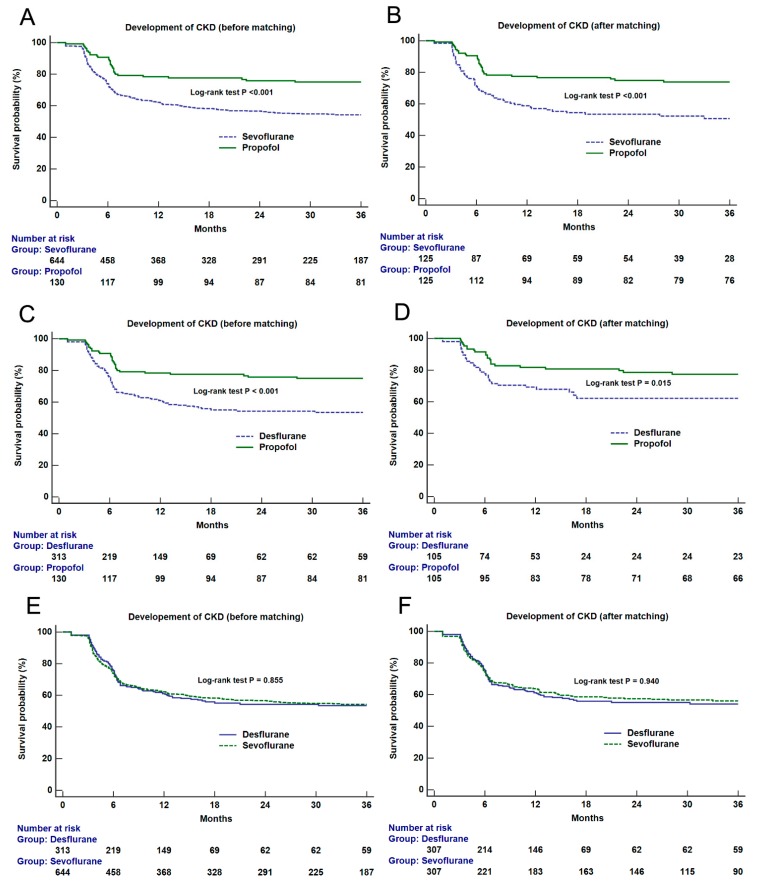
Kaplan-Meier survival curve analyses of new-onset chronic kidney disease stage 3a or high according to the main anesthetic agent groups (propofol vs. sevoflurane, upper, before (**A**) and after (**B**) matching; propofol vs. desflurane, middle, before (**C**) and after (**D**) matching; sevoflurane vs. desflurane, lower, before (**E**) and after (**F**) matching). The results of the log-rank test between groups are shown on the figure.

Kaplan-Meier survival analyses of the entire cohort showed significant differences in renal survival between the propofol and other volatile groups (Log-rank test: vs. sevoflurane, *p* < 0.001; vs. desflurane, *p* < 0.001) (Figure 2). After matching, there were significant differences in survival between the propofol and volatile agent groups (vs. sevoflurane, *p* < 0.001; vs. desflurane, *p* = 0.015) (Figure 2). However, no significant difference was observed between the sevoflurane and desflurane groups before and after matching (Figure 2). Regarding combined volatile group, there was a significant difference in renal survival between the propofol and volatile group (*p* < 0.001) (Supplementary Figure S5). The significant difference was also observed after matching (*p* = 0.032). 

The results of the subgroup analysis of partial nephrectomy were shown in Supplemental Table S2 and Supplemental Figures S6 and S7. We also performed propensity score matching in the subgroup and obtained 67 pairs of matched cases. No significant difference in the incidence of postoperative AKI or new-onset CKD was found between the propofol and volatile groups. However, the propofol group showed significantly less frequent postoperative CKD upstaging than the volatile group.

## 4. Discussion

We investigated the association between general anesthetic agents and postoperative renal functional outcomes in patients undergoing nephrectomy. The incidences of postoperative AKI and CKD upstaging were significantly and consistently lower in the propofol group compared to the sevoflurane or desflurane group before and after propensity score matching. The three-year postoperative incidence of new-onset CKD stage 3a or high was also significantly lower in the propofol group than the sevoflurane group after matching. There was no significant difference between sevoflurane and desflurane groups. Propofol was associated with better both short- and long-term renal function after nephrectomy compared to the volatile agents. However, subgroup analysis of partial nephrectomy did not show consistent results. Our results should be interpreted cautiously given the limitations of single-center retrospective design.

There was a significant association between the choice of general anesthetic agent and the incidence of AKI after nephrectomy in our study, favoring the propofol group. Several possible mechanisms can be elucidated on the basis of previous animal experiments. Propofol reduced postoperative AKI by attenuating oxidative stress in a rat model [26]. Propofol conferred a protective effect against renal ischemia-reperfusion injury by modulating inflammatory cytokines [27,28]. Considering the mechanisms of renal dysfunction after partial nephrectomy involves the ischemia-reperfusion injury by vascular clamping [29], propofol could be beneficial to attenuate AKI after nephrectomy. By reducing the incidence of AKI, propofol could attenuate the risk of CKD subsequently, as AKI is a potent risk factor of postoperative CKD [3,30]. 

The safety issue of sevoflurane has been raised since its introduction due to potential nephrotoxicity of its metabolite, compound A. However, despite the nephrotoxicity proven in an animal study, clinical studies demonstrated the safety of sevoflurane regarding renal function [19,31]. In a recent randomized trial conducted in patients undergoing kidney transplantation, there was no significant difference in graft outcome between the sevoflurane and propofol groups [32]. Conversely, previous animal studies reported the renal protective effect of sevoflurane [33,34]. However, to our knowledge, there was no previous animal or clinical study comparing propofol and sevoflurane during nephrectomy. The influence of anesthetic agent on renal function may be greater during nephrectomy with frequent and significant postoperative renal functional decline [13,35]. 

Recent studies reported results advocating propofol, which are consistent with our findings. A previous randomized study reported that the propofol-based anesthesia reduced the incidence of postoperative AKI compared to the sevoflurane group after valvular heart surgery [36]. They measured plasma inflammatory markers and suggested the reno-protective effect was mediated by the anti-inflammatory action of propofol. Propofol-based anesthesia reduced postoperative urinary kidney-specific proteins and serum pro-inflammatory cytokines compared to sevoflurane-based anesthesia in patients undergoing open abdominal aortic aneurysm repair [37]. In addition, in a retrospective study conducted on 4320 patients undergoing colorectal surgery, propofol decreased the incidence of postoperative AKI when compared to sevoflurane [38]. 

There were also studies reporting no effect of general anesthetic agents on postoperative renal function in other surgical populations. However, the number of studies which reported neutral effect was small. Although study design was different, a previous randomized trial showed no significant differences in renal function between sevoflurane, desflurane, and propofol after elective surgery [39]. However, this study involved only a small number of patients and did not limit the type of surgery. There was also no significant difference in the incidence of postoperative AKI after lung surgery between the propofol and sevoflurane in a recent retrospective study [40]. However, the incidence of AKI after lung surgery was as low as 3.5% and larger number of patients are required for sufficient study power. 

We performed propensity score analysis and used intraoperative vasopressor infusion as the contributor to the propensity score because vasopressor use could reflect intraoperative hypotension and was reported as an independent risk factor of postoperative AKI [41]. However, a recent retrospective study reported that intraoperative vasopressor infusion was not associated with AKI [42]. Vasopressor infusion during surgery could be a mediator to the development of AKI rather than a confounder because we infuse vasopressor to treat hypotension but vasopressor could also cause AKI [43].

The strength of our study is that we investigated the incidence of new-onset CKD after nephrectomy for 36 months after nephrectomy. Demographic and genetic factors, comorbidity, pre-existing renal disease, and surgical technique are associated with the development of CKD after nephrectomy [14]. However, there have been no reports of the association of anesthetic agents in the surgical population with long-term renal function. Although this was a single-center retrospective study, we demonstrated the possible benefit of propofol to mitigate the risk of CKD as well as AKI compared to volatile agents through rigorous adjustment of possible confounding factors. Matching was performed pairwise like a network analysis including matching for three different pairs of general anesthetics. The consistent results between different pair of network comparison supported our conclusion. However, we did not obtain significant difference in the incidence of AKI and new-onset CKD in the subgroup of partial nephrectomy. The incidence of AKI after partial nephrectomy is lower than the incidence after radical nephrectomy [11]. The incidences of AKI after partial and radical nephrectomy in our study were 16.0% and 58.0%, respectively. The incidences of new-onset CKD after partial and radical nephrectomy were 22.0% and 69.1%, respectively. As the incidences of AKI and CKD after partial nephrectomy are much lower than radical nephrectomy, a larger number of patients than our study would be required to detect any difference in the incidence of AKI or CKD after partial nephrectomy. Therefore, our subgroup analysis of partial nephrectomy may lack sufficient power. 

The results of our study should be interpreted cautiously under several limitations. First, it was a single-center retrospective analysis. Unmeasured or unknown confounders may have affected our study results. However, the pair-wise propensity score matching was used to minimize confounding. Sensitivity analyses of secondary outcomes yielded consistent results. Secondly, we did not exclude the patients with pre-existing CKD stage 3a or high not to decrease the study power. This might affect the incidence of AKI because preoperative CKD is known to be an important risk factor of postoperative AKI [44]. Furthermore, acute-on-chronic kidney injury is a different disease entity from AKI [45]. However, to minimize confounding by the baseline renal function, we used preoperative eGFR and serum albumin as contributors to the propensity score analysis. Furthermore, our secondary outcome of CKD upstaging could detect the renal functional decline even in the patients with baseline CKD. Thirdly, we used only serum creatinine concentration except urine output to diagnose the AKI. However, urine output criteria may be inaccurate due to mannitol infusion during partial nephrectomy [11]. Fourthly, we included all types of surgical approach, which was reported to be associated with postoperative AKI [10]. However, we could not perform the subgroup analysis according to the type of surgical approach due to the small number of patients in each surgical category. We only performed a propensity score analysis using the type of surgical approach and operation time as contributors. Fifthly, mannitol was routinely administered in our patients undergoing partial nephrectomy, but a recent randomized trial showed no effect of mannitol infusion on postoperative renal function [46]. However, since mannitol infusion did not affect postoperative eGFR [46] and we did not use the urine output criteria of AKI, the effect of mannitol on our study results would be insignificant. Sixthly, in the volatile groups, propofol bolus dose was used to induce anesthesia and this might have some residual effect in the volatile groups. However, propofol is distributed and eliminated rapidly after single bolus injection [47]. Therefore, the dose of propofol (1–2 mg/kg) used during anesthesia induction would not be a significant confounder for our analysis. Lastly, we performed post hoc power analysis instead of prior power analysis due to the limited number of patients. A sufficient number of patients were not included for the primary outcome according to our post hoc power calculation.

## 5. Conclusions 

In our propensity score-matched comparison of the general anesthetic agents in patients undergoing radical and partial nephrectomy, propofol was associated with a lower incidence of postoperative AKI and CKD upstaging compared to sevoflurane or desflurane. The three-year renal survival after nephrectomy was also significantly better for propofol compared to volatile agents. Therefore, in patients receiving nephrectomy, propofol may be the reasonable general anesthetic agent to mitigate postoperative renal functional deterioration compared to volatile agents. However, inconsistent subgroup analysis of partial nephrectomy and significant limitations of our study design preclude a firm conclusion. 

## Figures and Tables

**Table 1:** Patient characteristics and perioperative parameters.

Characteristics	Propofol (*n* = 130)	Sevoflurane (*n* = 644)	Desflurane (*n* = 313)	*p*-Value
**Demographic data**				
Age, years	55 (47–62)	57 (48–67)	58 (49–66)	0.12
Female, *n*	34 (26.2)	199 (30.9)	87 (27.8)	0.42
Body-mass index, kg/m²	24.5 (22.4–26.6)	24.5 (22.6–26.6)	24.5 (22.5–26.6)	0.98
Current smoker, *n*	22 (16.9)	85 (13.2)	59 (18.8)	0.06
**Background medical status**				
ASA 1/2/3/4	69 (53.1)/55 (42.3)/6 (4.6)/0	317 (49.2)/280 (43.5)/47 (7.3)/0	119 (38.0)/181 (57.8)/12 (3.8)/1 (0.3)	<0.001
Hypertension, *n*	46 (35.4)	292 (45.3)	131 (41.9)	0.10
Diabetes mellitus, *n*	16 (12.3)	99 (15.4)	53 (16.9)	0.47
Cerebrovascular accident, *n*	4 (3.1)	17 (2.6)	4 (1.3)	0.34
Liver disease, *n*	11 (8.5)	22 (3.4)	13 (4.2)	0.03
Ischemic heart disease, *n*	2 (1.5)	11 (1.7)	2 (0.6)	0.41
Hyperlipidemia, *n*	8 (6.2)	59 (9.2)	34 (10.9)	0.29
Preoperative eGFR (mL/min/1.73 m^2^)	82 (73–89)	81 (69–92)	77 (68–90)	0.068
Preoperative stage of CKD				0.09
1 (eGFR ≥ 90 mL/min/1.73 m^2^)	29 (22.3)	193 (30.0)	79 (25.2)	0.179
2 (60–89 mL/min/1.73 m^2^)	91 (71.0)	365 (56.7)	185 (59.1)	
3a (45–59 mL/min/1.73 m^2^)	4 (3.1)	47 (7.3)	29 (9.3)	
3b (30–44 mL/min/1.73 m^2^)	4 (3.1)	13 (2.0)	7 (2.2)	
4 (15–30 mL/min/1.73 m^2^)	1 (0.8)	3 (0.5)	2 (0.6)	
5 (< 15 mL/min/1.73 m^2^)	1 (0.8)	23 (3.6)	11 (3.5)	
Preoperative proteinuria, *n*	9 (6.9)	43 (6.7)	31 (9.9)	0.20
Preoperative hemoglobin, g/dL	14.3 (12.8–15.1)	13.7 (12.5–14.8)	13.8 (12.5–14.9)	0.04
Preoperative albumin, g/dL	4.4 (4.2–4.7)	4.4 (4.1–4.6)	4.4 (4.2–4.6)	0.20
ECOG performance status				0.001
0/1/2/3	121/6/3/0	536/82/23/2	285/25/1/2	
Clinical stage				
T 1a/1b	103 (79.2)/ 14 (10.8)	416 (64.6)/ 126 (19.6)	231 (73.8)/ 25 (8.0)	0.23
T 2a/2b	7 (5.4)/-	65 (10.1)/ 15 (2.3)	35 (11.2)/ 6 (1.9)	
T 3a/3b/3c	3 (2.3)/2 (1.5)/1 (0.8)	12 (1.9)/4 (0.6)/6 (0.9)	4 (1.3)/6 (1.9)/6 (1.9)	
N 0/1	129 (99.2)/ 1 (0.8)	616 (95.7)/ 28 (4.3)	298 (95.2)/ 15 (4.8)	0.10
M 0/1	123 (94.6)/ 7 (5.4)	619 (96.1)/ 25 (3.9)	303 (96.8)/ 10 (3.2)	0.30
**Operation and anesthesia related**				
Surgery type				0.07
Radical nephrectomy, *n*	44 (33.8)	286 (44.4)	139 (44.4)	
Partial nephrectomy, *n*	86 (66.2)	358 (55.6)	174 (55.6)	
Surgical approach				<0.001
Laparoscopic, *n*	10 (7.7)	130 (20.2)	51 (16.3)	
Hand-assisted laparoscopic, *n*	2 (1.5)	22 (3.4)	11 (3.5)	
Robot-assisted, *n*	62 (47.7)	40 (6.2)	32 (10.2)	
Open, *n*	56 (43.1)	452 (70.2)	219 (70.0)	
Operation time, hour	2.8 (2.3–3.3)	2.2 (1.7–2.8)	2.2 (1.7–2.9)	<0.001
Renal ischemic time, min *	27 (21.5–35.5)	24.6 (20.0–31.0)	22.4 (17.4–27.5)	<0.001
Ischemia type *				0.13
Cold ischemia	1 (1.2)	22 (6.1)	7 (4.0)	
Warm ischemia	85 (98.8)	336 (93.9)	167 (96.0)	
Intraoperative vasopressor use, *n*	5 (3.8)	10 (1.6)	7 (2.2)	0.55
pRBC transfusion, *n*	15 (11.5)	68 (10.6)	40 (12.8)	0.59
Crystalloid administration, mL/kg	18.8 (12.3–24.9)	18.7 (14.2–25.0)	18.1 (12.5–25.2)	0.17
Colloid administration, mL/kg	0 (0–5.8)	0 (0–5.0)	0 (0–5.5)	0.78

The values are presented as the median (interquartile range) or number (%). * These values are for only partial nephrectomy. Liver disease includes hepatitis or liver cirrhosis. ASA = American society of Anesthesiologist physical classification, CKD = chronic kidney disease, ECOG performance status = Eastern Cooperative Oncology Group performance status, eGFR = estimated glomerular filtration rate, pRBC = packed red blood cell.

**Table 2:** Comparison of incidence of primary and secondary outcomes between patients according to the main anesthetic agents during surgery before and after propensity score matching.

Outcomes	Propofol	Sevoflurane	Risk Difference, %	*p*-Value
Number of patients before matching	130	644		
Postoperative AKI, *n*	29 (22.3)	229 (35.6)	−13 (−5 to −21)	0.032
Stage 1	24 (18.5)	203 (31.5)	−13 (−5 to −21)	
Stage 2	1 (0.8)	2 (0.3)	0.5 (−1.1 to 2.0)	
Stage 3	4 (3.1)	24 (3.7)	−0.6 (−3.9 to 2.7)	
Postoperative new-onset CKD stage 3a or high, *n*	33 (25.4)	296 (46.0)	−21 (−12 to −29)	<0.001
CKD upstaging, *n*	38 (29.2)	307 (47.7)	−18 (−10 to −27)	<0.001
Number of patients after matching	125	125		
Postoperative AKI, *n*	29 (23.2)	50 (39.5)	−17 (−5 to −28)	0.004
Stage 1	24 (19.2)	45 (36.0)	−17 (−6 to −28)	
Stage 2	1 (0.8)	-	-	
Stage 3	4 (3.2)	5 (4.0)	−0.8 (−5.4 to 3.8)	
Postoperative new-onset CKD stage 3a or high, *n*	33 (26.4)	61 (48.8)	−22 (−11 to −34)	<0.001
CKD upstaging, *n*	34 (27.2)	73 (58.4)	−31 (−20 to −43)	<0.001

The values are presented as the median (interquartile range) or number (%). AKI = acute kidney injury determined by KDIGO creatinine criteria, CKD = chronic kidney disease.

**Table 3:** Comparison of incidence of primary and secondary outcomes between patients according to the main anesthetic agents during surgery before and after propensity score matching.

Outcomes	Propofol	Desflurane	Risk Difference, %	*p*-Value
Number of patients before matching	130	313		
Postoperative AKI, *n*	29 (22.3)	113 (36.1)	−14 (−5 to −22)	0.042
Stage 1	24 (18.5)	100 (31.9)	−13 (−5 to −22)	
Stage 2	1 (0.8)	3 (1.0)	−0.2 (−2.0 to 1.7)	
Stage 3	4 (3.1)	10 (3.2)	−0.1 (−3.7 to 3.4)	
Postoperative new-onset CKD stage 3a or high, *n*	33 (25.4)	131 (41.9)	−16 (−7 to −26)	0.001
CKD upstaging, *n*	38 (29.2)	141 (45.0)	−16 (−6 to −25)	0.002
Number of patients after matching	105	105		
Postoperative AKI, *n*	22 (21.0)	36 (34.3)	−13 (−1 to −25)	0.031
Stage 1	19 (18.1)	31 (29.5)	−11 (−0.1 to −23)	
Stage 2	1 (1.0)	-	-	
Stage 3	2 (1.9)	5 (4.8)	−2.9 (−7.7 to 2.0)	
Postoperative new-onset CKD stage 3a or high, *n*	24 (22.9)	35 (33.3)	−10 (−23 to 2)	0.09
CKD upstaging, *n*	34 (32.4)	51 (48.6)	−16 (−29 to −3)	0.017

The values are presented as the median (interquartile range) or number (%). AKI = acute kidney injury determined by KDIGO creatinine criteria, CKD = chronic kidney disease.

**Table 4:** Comparison of incidence of primary and secondary outcomes between patients according to the main anesthetic agents during surgery before and after propensity score matching.

Outcomes	Sevoflurane	Desflurane	Risk Difference, %	*p*-Value
Number of patients before matching	644	313		
Postoperative AKI, *n*	229 (35.6)	113 (36.1)	−0.5 (−7.0 to 5.9)	0.98
Stage 1	203 (31.5)	100 (31.9)	−0.4 (−6.7 to 5.9)	
Stage 2	2 (0.3)	3 (1.0)	−0.6 (−1.8 to 0.5)	
Stage 3	24 (3.7)	10 (3.2)	0.5 (−1.9 to 3.0)	
Postoperative new-onset CKD stage 3a or high, *n*	296 (46.0)	131 (41.9)	4.1 (−2.6 to 10.8)	0.23
CKD upstaging, *n*	307 (47.7)	141 (45.0)	2.7 (−4.1 to 9.4)	0.45
Number of patients after matching	307	307		
Postoperative AKI, *n*	103 (33.6)	110 (35.8)	−2.3 (−9.8 to 5.2)	0.55
Stage 1	93 (30.3)	99 (32.2)	−2.0 (−9.3 to 5.4)	
Stage 2	-	3 (1.0)	-	
Stage 3	10 (3.3)	8 (2.6)	0.7 (−2.0 to 3.3)	
Postoperative new-onset CKD stage 3a or high, *n*	136 (44.3)	127 (41.4)	2.9 (−4.9 to 10.8)	0.46
CKD upstaging, *n*	147 (47.9)	139 (45.3)	2.6 (−5.3 to 10.5)	0.52

The values are presented as the median (interquartile range) or number (%). AKI = acute kidney injury determined by KDIGO creatinine criteria, CKD = chronic kidney disease.

## Supplementary Materials

The following are available online at https://www.mdpi.com/2077-0383/8/10/1530/s1, Table S1: Comparison of incidence of primary and secondary outcomes between patients according to the main anesthetic agents (propofol vs. volatile agents) during surgery before and after propensity score matching, Table S2: Comparison of incidence of primary and secondary outcomes between patients according to the main anesthetic agents (propofol vs. volatile agents) before and after propensity score matching in the subgroup of partial nephrectomy, Figure S1: Histograms (left) and covariate balance plot (right) of distribution of standardized differences of covariates between the patients who received propofol and sevoflurane during surgery in all cohort, Figure S2: Histograms (left) and covariate balance plot (right) of distribution of standardized differences of covariates between the patients who received propofol and desflurane during surgery in all cohort, Figure S3: Histograms (left) and covariate balance plot (right) of distribution of standardized differences of covariates between the patients who received sevoflurane and desflurane during surgery in all cohort, Figure S4: Histograms (left) and covariate balance plot (right) of distribution of standardized differences of covariates between the patients who received propofol and volatile agents during surgery in all cohort, Figure S5: Kaplan-Meier survival curve analysis of new-onset chronic kidney disease according to the main anesthetic agents (TIVA vs. volatile agents) before (A) and after (B) propensity score matching in all cohort, Figure S6: Histograms (left) and covariate balance plot (right) of distribution of standardized differences of covariates between the patients who received propofol and volatile agents during surgery in the subgroup of partial nephrectomy, Figure S7: Kaplan-Meier survival curve analysis of new-onset chronic kidney disease according to the main anesthetic agents (TIVA vs. volatile agents) before (A) and after (B) propensity score matching in the subgroup of partial nephrectomy.
