# Supplementary material for: General Anesthetic Agents and Renal Function after Nephrectomy
Source: J Clin Med. 2019 Sep 24;8(10):1530. doi: 10.3390/jcm8101530 (PMC6832234; doi:10.3390/jcm8101530)

General anesthetic agents and renal function after nephrectomy

: Supplemental Materials

Number	Title	Page
**Supplemental Table S1.**	Comparison of incidence of primary and secondary outcomes between patients according to the main anesthetic agents (propofol vs. volatile agents) during surgery before and after propensity score matching.	2
**Supplemental Table S2.**	Comparison of incidence of primary and secondary outcomes between patients according to the main anesthetic agents (propofol vs. volatile agents) before and after propensity score matching in the subgroup of partial nephrectomy.	3
**Supplemental Figure S1.**	Histograms (left) and covariate balance plot (right) of distribution of standardized differences of covariates between the patients who received propofol and sevoflurane during surgery in all cohort.	4
**Supplemental Figure S2.**	Histograms (left) and covariate balance plot (right) of distribution of standardized differences of covariates between the patients who received propofol and desflurane during surgery in all cohort.	5
**Supplemental Figure S3.**	Histograms (left) and covariate balance plot (right) of distribution of standardized differences of covariates between the patients who received sevoflurane and desflurane during surgery in all cohort.	6
**Supplemental Figure S4.**	Histograms (left) and covariate balance plot (right) of distribution of standardized differences of covariates between the patients who received propofol and volatile agents during surgery in all cohort.	7
**Supplemental Figure S5.**	Kaplan-Meier survival curve analysis of new-onset chronic kidney disease according to the main anesthetic agents (TIVA vs. volatile agents) before (A) and after (B) propensity score matching in all cohort.	8
**Supplemental Figure S6.**	Histograms (left) and covariate balance plot (right) of distribution of standardized differences of covariates between the patients who received propofol and volatile agents during surgery in the subgroup of partial nephrectomy.	9
**Supplemental Figure S7.**	Kaplan-Meier survival curve analysis of new-onset chronic kidney disease according to the main anesthetic agents (TIVA vs. volatile agents) before (A) and after (B) propensity score matching in the subgroup of partial nephrectomy.	10

Supplemental Table S1. Comparison of incidence of primary and secondary outcomes between patients according to the main anesthetic agents (propofol vs. volatile agents) during surgery before and after propensity score matching.

Outcomes	Propofol	Volatile agents	Risk difference, %	P-value
Number of patients before matching	130	957		
Postoperative AKI, n	29 (22.3)	342 (35.7)	-13 (-6 to -21)	0.002
Stage 1	24 (18.5)	303 (31.7)	-13 (-6 to -20)	
Stage 2	1 (0.8)	5 (0.5)	0.2 (-1.3 to 1.8)	
Stage 3	4 (3.1)	34 (3.6)	-0.5 (-3.7 to 2.7)	
Postoperative new-onset CKD stage 3a, n	33 (25.4)	427 (44.6)	-19 (-11 to -27)	<0.001
CKD upstaging, n	38 (29.2)	448 (46.8)	-18 (-9 to -26)	<0.001
Number of patients after matching	126	126		
Postoperative AKI, n	27 (21.4)	43 (34.1)	-13 (-2 to -24)	0.024
Stage 1	23 (18.3)	41 (32.5)	-14 (-4 to -25)	
Stage 2	1 (0.8)	-	-	
Stage 3	3 (2.4)	2 (1.6)	0.8 (-3.2 to 4.0)	
Postoperative new-onset CKD stage 3a, n	32 (25.4)	44 (34.9)	-10 (-21 to 2)	0.100
CKD upstaging, n	36 (28.6)	69 (54.8)	-26 (-38 to -14)	<0.001

The values are presented as the median (interquartile range) or number (%).

AKI = acute kidney injury determined by KDIGO creatinine criteria, CKD = chronic kidney disease.

Supplemental Table S2. Comparison of incidence of primary and secondary outcomes between patients according to the main anesthetic agents (propofol vs. volatile agents) before and after propensity score matching in the subgroup of partial nephrectomy.

Outcomes	Propofol	Volatile agents	Risk difference, %	P-value
Number of patients before matching	86	532		
Postoperative AKI, n	13 (15.1)	86 (16.2)	-10.5 (-9.2 to 7.1)	0.806
Stage 1	9 (10.5)	80 (15.0)	-4.6 (-11.7 to 2.6)	
Stage 2	1 (1.2)	2 (0.4)	0.8 (-1.5 to 3.1)	
Stage 3	3 (3.5)	4 (0.8)	2.7 (-1.2 to 6.7)	
Postoperative new-onset CKD stage 3a, n	15 (17.4)	121 (22.7)	-5.3 (-14.1 to 3.5)	0.271
CKD upstaging, n	29 (33.7)	246 (46.2)	-12.5 (-1.7 to 23.4)	0.030
Number of patients after matching	67	67		
Postoperative AKI, n	12 (17.9)	14 (20.9)	-3.0 (-16.4 to 10.4)	0.663
Stage 1	8 (11.9)	12 (17.8)	-6.0 (-18.0 to 6.1)	
Stage 2	1 (1.5)	1 (1.5)	0.0 (-4.1 to 4.1)	
Stage 3	3 (4.5)	2 (1.5)	1.5 (-5.0 to 7.9)	
Postoperative new-onset CKD stage 3a, n	12 (17.9)	15 (22.4)	-4.5 (-18.0 to 9.1)	0.520
CKD upstaging, n	25 (37.3)	37 (55.2)	-17.9 (-34.5 to -1.3)	0.038

The values are presented as the median (interquartile range) or number (%).

AKI = acute kidney injury determined by KDIGO creatinine criteria, CKD = chronic kidney disease.

Supplemental Figure S1. Histograms (left) and covariate balance plot (right) of distribution of standardized differences of covariates between the patients who received propofol and sevoflurane during surgery in all cohort. BMI = body-mass index, ASA_class = American Society of Anesthesiologist physical status classification, HTN = hypertension, DM = diabetes mellitus, CVA = cerebrovascular accident, Hb = hemoglobin, Alb = albumin, GFR = glomerular filtration rate, pRBC = packed red blood cells.

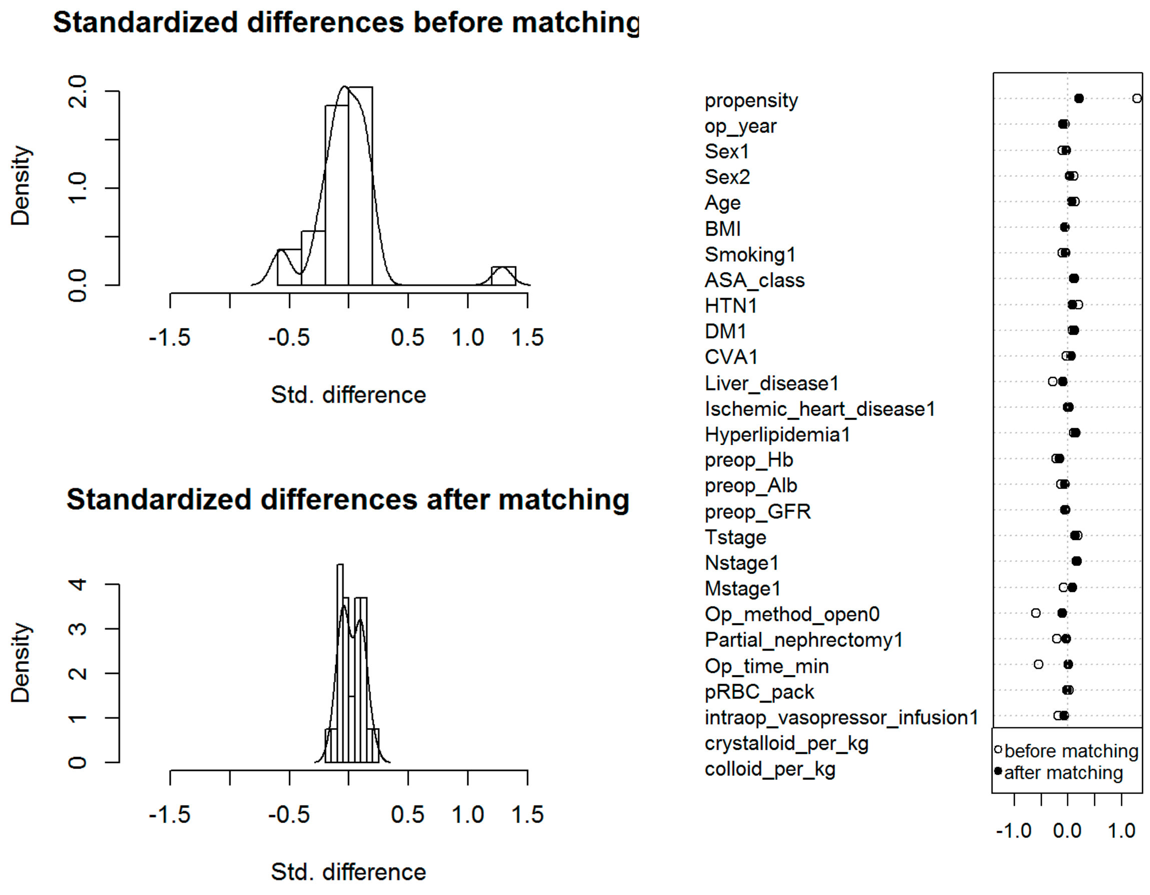

Supplemental Figure S2. Histograms (left) and covariate balance plot (right) of distribution of standardized differences of covariates between the patients who received propofol and desflurane during surgery in all cohort. BMI = body-mass index, ASA_class = American Society of Anesthesiologist physical status classification, HTN = hypertension, DM = diabetes mellitus, CVA = cerebrovascular accident, Hb = hemoglobin, Alb = albumin, GFR = glomerular filtration rate, pRBC = packed red blood cells.

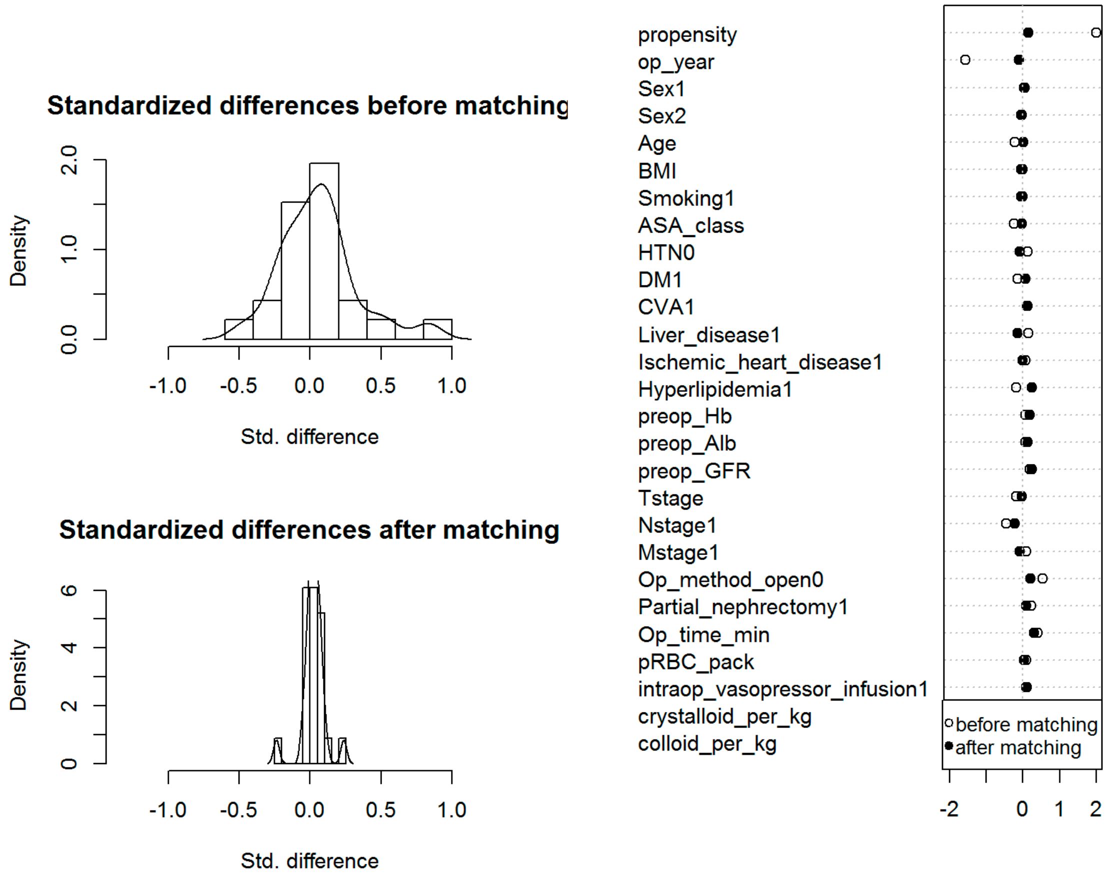

Supplemental Figure S3. Histograms (left) and covariate balance plot (right) of distribution of standardized differences of covariates between the patients who received sevoflurane and desflurane during surgery in all cohort. BMI = body-mass index, ASA_class = American Society of Anesthesiologist physical status classification, HTN = hypertension, DM = diabetes mellitus, CVA = cerebrovascular accident, Hb = hemoglobin, Alb = albumin, GFR = glomerular filtration rate, pRBC = packed red blood cells.

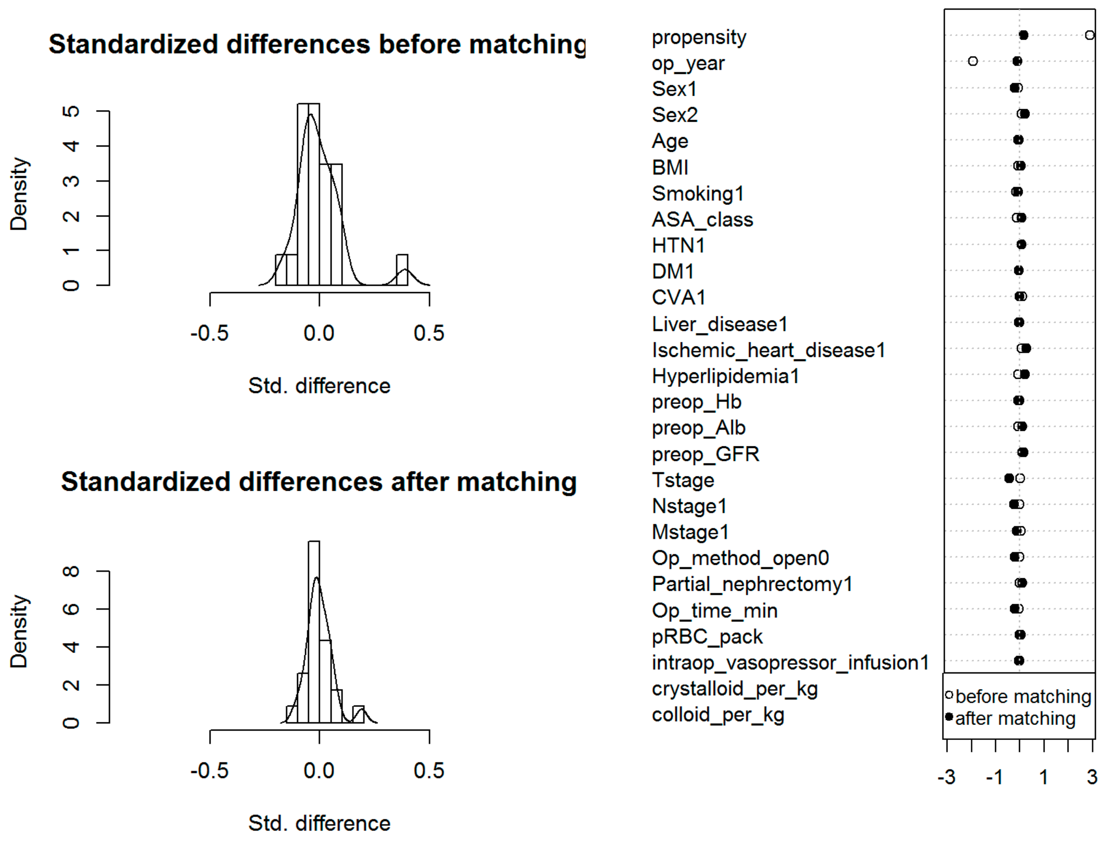

Supplemental Figure S4. Histograms (left) and covariate balance plot (right) of distribution of standardized differences of covariates between the patients who received propofol and volatile agents during surgery in all cohort. BMI = body-mass index, ASA_class = American Society of Anesthesiologist physical status classification, HTN = hypertension, DM = diabetes mellitus, CVA = cerebrovascular accident, Hb = hemoglobin, Alb = albumin, GFR = glomerular filtration rate, pRBC = packed red blood cells.

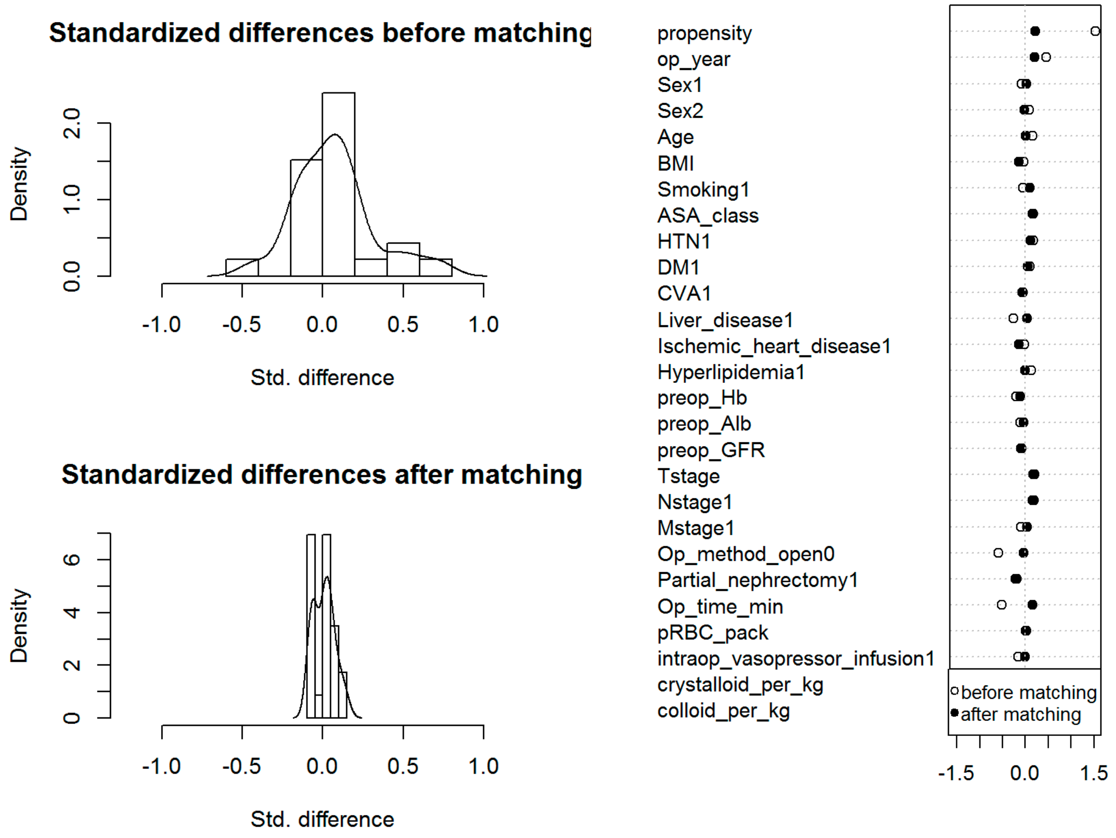

Supplemental Figure S5. Kaplan-Meier survival curve analysis of new-onset chronic kidney disease according to the main anesthetic agents (TIVA vs. inhalation agents) before (A) and after (B) propensity score matching in all cohort. The results of log-rank test between the groups are shown on the figure.

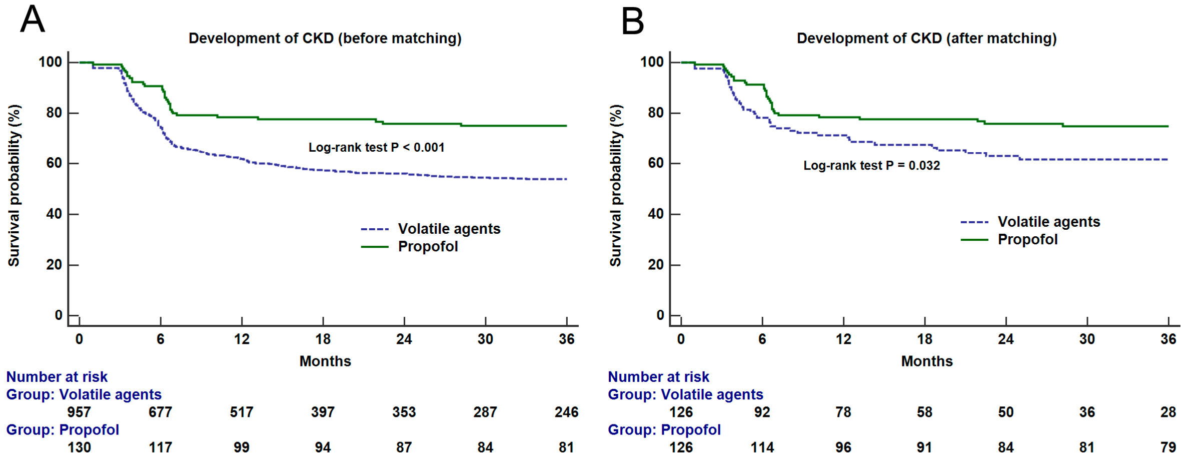

Supplemental Figure S6. Histograms (left) and covariate balance plot (right) of distribution of standardized differences of covariates between the patients who received propofol and volatile agents during surgery in the subgroup of partial nephrectomy. BMI = body-mass index, ASA_class = American Society of Anesthesiologist physical status classification, HTN = hypertension, DM = diabetes mellitus, CVA = cerebrovascular accident, Hb = hemoglobin, Alb = albumin, GFR = glomerular filtration rate, pRBC = packed red blood cells.

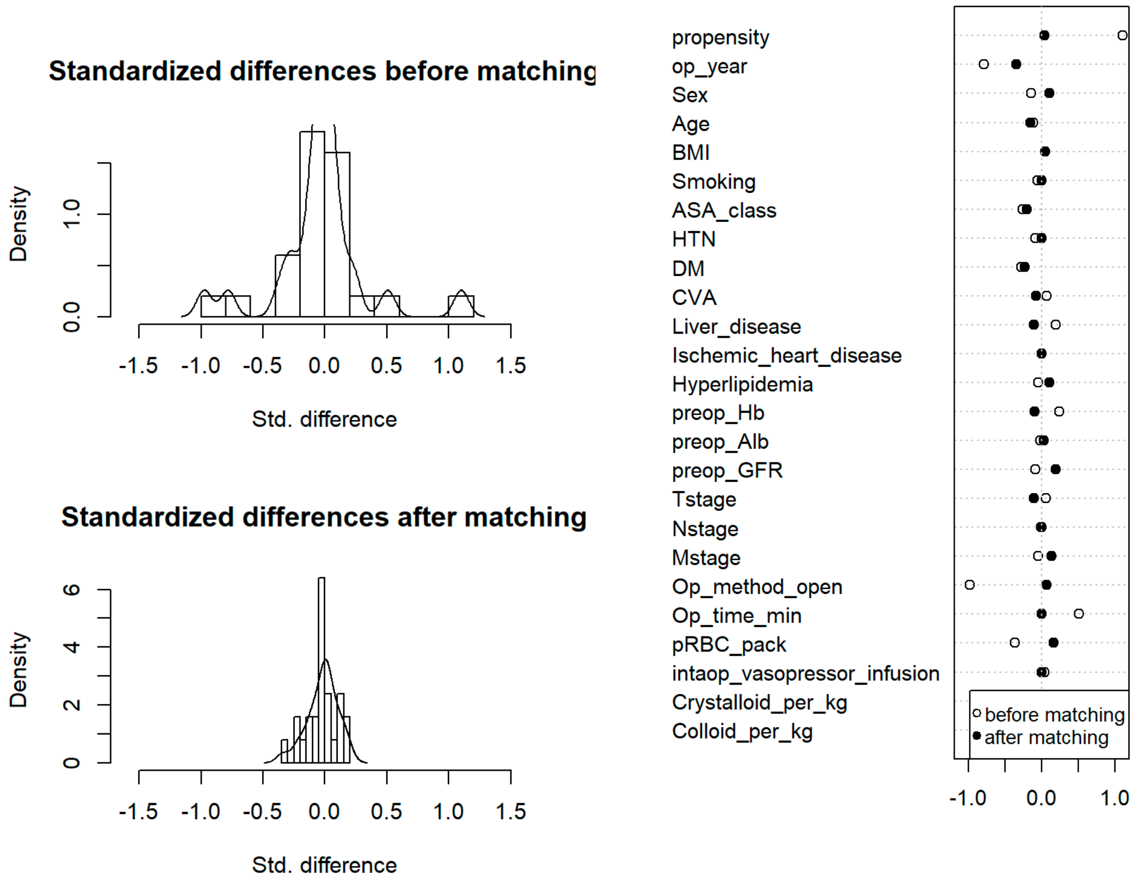

Supplemental Figure S7. Kaplan-Meier survival curve analysis of new-onset chronic kidney disease according to the main anesthetic agents (TIVA vs. inhalation agents) before (A) and after (B) propensity score matching in the subgroup of partial nephrectomy. The results of log-rank test between the groups are shown on the figure.

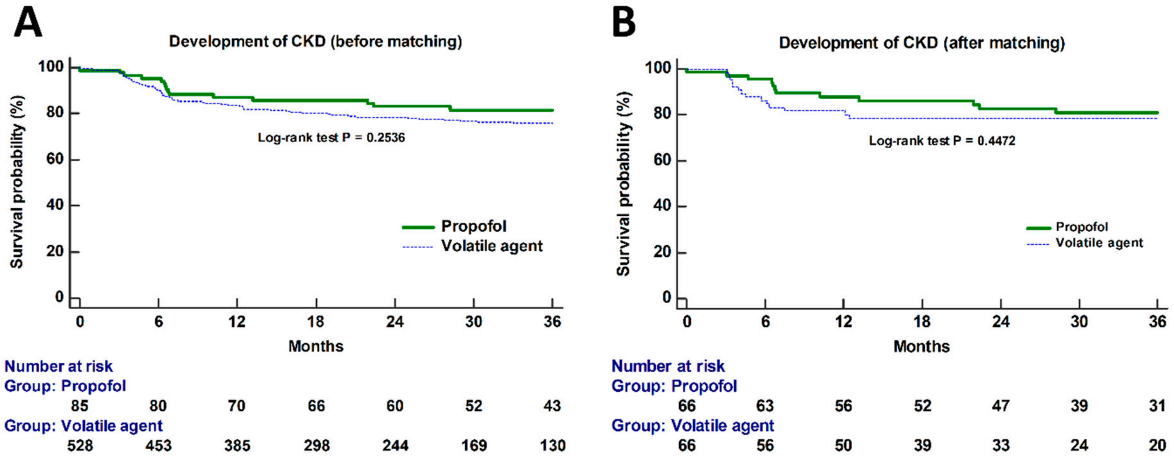

Supplement: Supplementary file 1 [file jcm-08-01530-s001.pdf]
